# Gauging climate preparedness to inform adaptation needs: local level adaptation in drinking water quality in CA, USA

**DOI:** 10.1007/s10584-016-1870-3

**Published:** 2016-12-23

**Authors:** Julia A. Ekstrom, Louise Bedsworth, Amanda Fencl

**Affiliations:** Policy Institute for Energy, Environment and the Economy, University of California Davis, Davis, CA USA

## Abstract

Understanding resource managers’ perceptions of climate change, analytic capacity, and current adaptation activities can provide insight into what can help support adaptation processes at the local level. In California, where a major drought currently demonstrates some of the hardships that could be regularly encountered under a changing climate, we present results from a survey of drinking water utilities about the perceived threat, analytic capacity, and adaptation actions related to maintaining water quality in the face of climate change. Among surveyed utilities (*n* = 259), awareness is high in regard to climate change occurring and its potential impacts on water quality globally, but perceived risk is lower with regard to climate impacts on *local* drinking water quality. Just over half of surveyed utilities report at least some adaptation activity to date. The top three variables that most strongly correlated with reported adaptation action were (1) perceived risk on global and local water quality, (2) surface water reliance, and (3) provision of other services beyond drinking water. Other tested variables significantly correlated with reported adaptation action were (4) degree of impact from the current drought and (5) communication with climate change experts. Findings highlight that smaller groundwater-reliant utilities may need the most assistance to initiate climate adaptation processes. Trusted information sources most frequently used across respondents were state government agencies, followed by colleagues in the same utilities. The finding that frequently used sources of information are similar across utilities presents a promising opportunity for training and disseminating climate information to assist those systems needing the most support.

**Electronic supplementary material:**

The online version of this article (doi:10.1007/s10584-016-1870-3) contains supplementary material, which is available to authorized users.

## Introduction

**Fig. 1:**
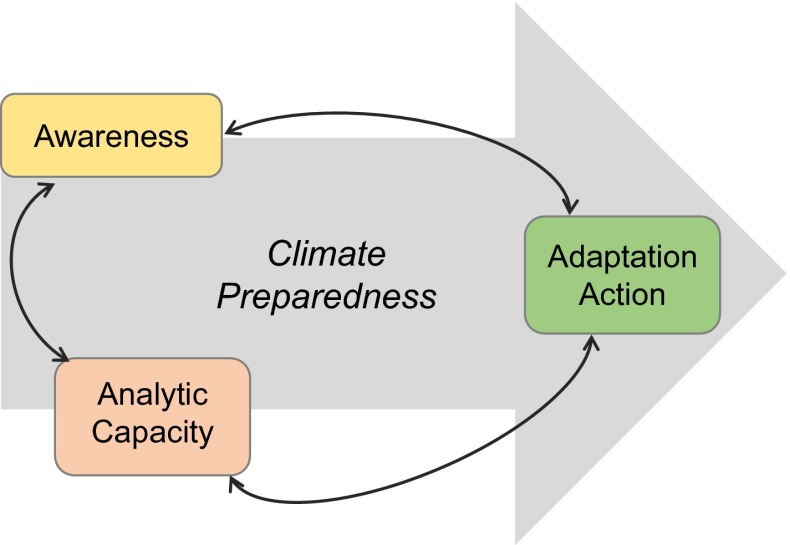
Three important and inter-related dimensions for understanding how prepared resource managers are for climate change impacts (based off the framework developed by Moser and Luers 2008)

This article builds on a growing body of work to build a better understanding of the needs of resource managers to prepare for a changing climate (Rayner et al. 2005; Moser and Tribbia 2007; Bedsworth and Hanak 2010; Mastrandrea et al. 2010; Archie et al. 2014; Kiem et al. 2015). Top-down approaches attempting to define managers’ needs have tried to fill the “information-deficit” with more science as prioritized by scientists (Moser and Dilling 2007). In contrast, the heart of the bottom-up approach involves understanding resource managers’ concerns, perspectives (including their trust of different sources), regulatory requirements, organizational norms, and decision-making processes. Here, we focus on understanding the perceptions, capacities, and climate adaptation efforts of drinking water quality managers (Fig. 1). Together, these shed light on where assistance or guidance may be useful to support future adaptation (Moser and Luers 2008). At the same time, we create a baseline of perceptions and actions statewide that can be monitored and evaluated over time as adaptation is mainstreamed.

## Study location

California has a complex water delivery system to serve its nearly 39 million residents in a Mediterranean climate of dry summers and wet winters. Much of the system’s supplies originates in the state’s upper watersheds and then extends through organized natural and built conveyances to bring water to taps in homes, businesses, and farms. Previous studies have shown the vulnerability of California’s water supply to a changing climate. Increasing temperatures and shifting precipitation patterns are expected to reduce snowpack, a critical water storage reservoir for the state (Cayan et al. 2012). California is currently in its fifth consecutive year of an historic drought, leading the Governor to declare a State of Emergency and require consumption reductions for urban water systems statewide (Brown 2015). Therefore, drought has highlighted the vulnerability of this system and likely shapes the perspectives and perceptions of our research subjects.

California is a prime geography for this initial survey because of its climate leadership, investment in understanding climate risk, especially to the water system, and its rapidly expanding integration of adaptation and resilience into its climate policy. California is a proven leader in environmental policy, which in the past decade has included climate change. California adopted the nation’s first climate mitigation statute in 2006, The Global Warming Solutions Act (California Health and Safety Code, Division 25.5), and has required regular assessments of the projected impacts of climate change on the state since 2005 (Franco et al. 2011; Moser et al. 2012), and in 2015, it began requiring consideration of climate impacts in local government planning (California Government Code Section 65302). Further laws and executive actions direct state agencies to consider climate change in all planning and investment.

### Focus on quality

The majority of climate change analyses on water have focused on the impacts on quantity, rather than *quality* (Michalak 2016). However, the quality of California’s water, in groundwater aquifers and surface water (lakes, reservoirs, streams, and rivers—including the coastal-influenced Sacramento-San Joaquin Delta), already suffer with extreme weather events and is expected to degrade with climate change (Bates et al. 2008; Anderson et al. 2009; Delpla et al. 2009; Boehlert et al. 2015).

### Climate change impacts

Climate change is expected to affect the quality of drinking water through several pathways (Pal et al. 2015; Boehlert et al. 2015; Khan et al. 2015). Increased runoff from extreme precipitation can contribute to pollution of rivers, streams, and other water bodies, contaminating supplies and creating more challenges for drinking water treatment (Romero-Lankao et al. 2014). Increasing temperatures can lead to more algal and bacterial blooms, which can complicate treatment or even create toxic conditions from direct exposure, as experienced in 2015 in Napa and Contra Costa Counties. As conservation measures increase, some utilities may rely on longer storage times, which can also lead to disinfectant problems. Wildfires, projected to increase (Westerling and Bryant 2008), pose a threat to water quality by degrading watersheds of source water, contributing to increased turbidity through runoff and water pollution (Emelko et al. 2011), and jeopardizing treatment infrastructure.

Groundwater is also at risk from projected increases in severity and frequency of droughts (Scanlon et al. 2012). In addition to increasing demand on groundwater, drought can threaten quality of groundwater by concentrating existing contaminants, shifting contaminants closer to well intakes, and shifting contaminant plumes between connected aquifers. The anticipated acceleration of the rate of sea level rise can exacerbate coastal storm and flood risks by increasing salt water intrusion into coastal aquifers and estuaries, raising salinity levels in drinking water supplies and impacting water quality infrastructure, such as delta levee systems (Chen et al. 2010).

### Utilities in adaptation

For over 98% of the population in California, water utilities will be the frontline responder to climate change impacts on drinking water quality. Utilities are responsible for providing clean drinking water to residents (EPA 1974), and at the same time, they determine the affordability for customers, challenges that are likely to become more difficult with climate change (Christian-Smith et al. 2013; Hanak et al. 2014).

A utility’s given reliance on surface water and/or groundwater supplies depends on where they are located; their local demand, water rights, and availability; and type of water year—dry, average, or wet (SWRCB 2015). Depending on the utility, surface water is locally sourced and/or imported from within or beyond California. Groundwater basins throughout California serve as the other primary source of drinking water, which are tapped into through both shallow and deep wells by utilities and individual households, irrigation districts, and industries.

### Legal requirements to adapt

Thus far, no federal legal requirement or incentive structure exists for water utilities to prepare for climate change impacts on drinking water quality. At the state level, documenting the vulnerability of systems is a part of *recommended* planning for larger utilities (Conrad 2013) and for those that choose to participate in a regional planning process (Conrad 2012). Adaptation of these utilities, therefore, depends largely on progressive voluntary actions, which requires extended effort and leadership to self-fund and navigate through adaptation processes. State directives around Urban Water Management Plans (UWMP) and its encouragement of Integrated Regional Water Management Plans (IRWMP) support consideration of climate change, but neither requires it.

To understand where these efforts have (and have not) arisen, we investigate how prepared drinking water utilities are for climate change impacts on water quality in California. Based on the framework developed by Moser and Luers (2008), we use three dimensions to gauge climate preparedness: climate awareness, analytic capacity, and adaptation activity. Then, we explore significant variables of utilities that may contribute to impeding climate adaptation activity.

## Methods

We collected baseline information about the dimensions of climate preparedness using an online survey in July and August 2015 of drinking water utilities (districts, municipalities, etc.). This survey asked utilities questions about existing water quality threats, perceptions of climate change, climate adaptation activities, and information uses. Prior to developing and testing the survey, we conducted initial unstructured, informative meetings with utilities, state and federal water managers, document review/analysis (water management plans), and data inquiry/collection from the State Water Resources Control Board (SWRCB) and the Department of Water Resources (DWR).

### Survey of utilities—structure

We organized the flow of the survey instrument so that it was in the sequence of a set of conversation topics, beginning with collecting background and verification information about the respondent and his/her utility, followed by water quality impact questions and then leading to climate change questions. The instrument included six major sections (Supplemental Information contains survey questions):

- Background information
- Current water quality threats
- Current drought impacts on water quality
- Awareness of climate change
- Perceived threat of climate change
- Information sources and needs

We constructed survey questions using a combination of closed- and open-ended questions. Closed-ended questions allowed collection of a respondent’s opinions on a quantitative scale, whereas open-ended questions allowed respondents to express precisely their opinions and experience. The survey was conducted online (using Qualtrics), so that we were able to include a panel of background information about respondents that allowed for survey customization depending on the recipient’s water supply portfolio.

### Targeted population

Due to limited data availability and complex regulatory circumstances, development of a survey population was a challenging task that had to balance inclusiveness, but also availability of data to allow for meaningful analysis of survey results. The State Water Resources Control Board (SWRCB) and the Department of Water Resources (DWR) provided contact details (name, email, title, and utility affiliation) for select populations of water utilities, from which we constructed the survey respondent list. The SWRCB provided contact information and data for each public water system (utility) that submitted the 2014 Annual Report form, as required by the SWRCB to implement the California Safe Drinking Water Act. Given that the list contains only half of public water systems (and many were duplicates or contained NULL values), we supplemented the contact list with the points of contact for the Urban Water Management Plan (UWMP) for each public water system(s) available from the DWR.^1^ UWMPs are required for all urban water suppliers that provide over 3000 acre-feet of water annually or that serve more than 3000 connections. From these lists, we developed a survey population that included systems of all sizes with 200 or more service connections. This service connection threshold was chosen to align with the utilities that fall under the SWRCB’s direct regulatory authority.^2^ The majority of these systems are classified as community water systems, which provide water to residences. The majority of systems with <200 services connections provide water to non-residential facilities (Table S2).

The final respondent pool was constructed from this list of systems with greater than 200 connections. To enable meaningful analysis of survey responses, we further reduced the list to include utilities with water supply source data. Our final target population was 756 respondents representing 925 public water systems (utilities) that distribute drinking water through 8.1 million potable connections. Therefore, of the 2915 public water systems with ≥200 service connections in California, we were able to get contact information for 32% of these (for more detail on each step in the development of the survey population, see the Supplementary Information). As a result of the data limitations and resultant sample size, the surveyed population is not likely to be generalizable to all water utilities. However, we feel that it provides a meaningful initial study population and can provide helpful direction for future research.

### Preparedness indicators

To summarize the climate awareness, analytic capacity, and activity across survey responses, we developed simple sub-indices to gauge the relative extent of these three dimensions. Eleven variables represented these three dimensions.

To compute the score for *Awareness*, we combined four variables collected from the survey. These included agreement (1) or lack of agreement (neutral or disagreement, 0) that climate change is happening globally and locally (Table 1, nos. 1 and 3) and agreement or lack of agreement that climate change is or will affect water quality globally and locally (Table 1, nos. 2 and 4). We calculated the standardized mean of the four variables to compare across each respondent. We similarly computed *Analytic Capacity* and *Adaptation Action* across four and three variables, respectively (Table 1).

**Table 1:**
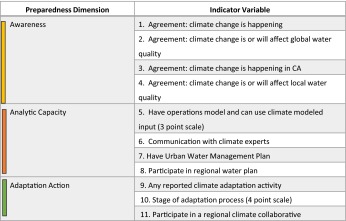
Indicators used to gauge three dimensions of climate preparedness of surveyed water utilities. All metrics were dichotomous, unless noted with other scoring in *parentheses*. See Supplemental Information for scale definitions and normalization

## Results

This section reports the response rate and survey population, followed by the overall findings of the current threats to water quality facing utilities, varying perceptions of climate change, climate adaptation efforts, and climate adaptation strategies pursued.

### Survey population and response rate

Partial responses were included in the response rate count and analysis. We included survey responses in our analysis if the respondents answered at least one question beyond the background information section. Response rates in this section were calculated based on the American Association for Public Opinion Research’s 2015 Standard Definitions for Response Rate 2, meaning that we include partial answers (AAPOR 2015).

In summary, out of the 756 water utilities that we invited to participate in the survey, we received 259 responses giving us a response rate of 34%. The pool of respondents represented a total of 11% of all public water systems with ≥200 service connections in California (311/2915, see Supplementary Information Tables S1–S8). The response rates by water supply portfolio (Supplemental Tables S6–8) ranged between 28 and 45%, which were similar to the overall response rate, but response rates were not as consistent by size (Table S8). The smallest systems were proportionally under-represented whereas the largest systems were over-represented (Table S8). Underlying capacity constraints may explain at least part of this disproportion of responses by size. We recognize the limited generalizability of the responses, but we hypothesize that our responses could be coming from the most advanced in climate adaptation and we are likely not seeing the least prepared utilities.

### Water quality and extreme events

Summarized below, responses to questions about water quality threats and then extreme weather events verified the clear and extant relationship between water quality and extreme weather and climatic events experienced by respondents.

In terms of *surface water*, all 95 respondents identified one or more ways that their surface water quality threats are triggered or worsened by weather or other environmental events (100% who saw and responded to the question). The threats that these respondents linked to weather events^3^ were point source pollution, non-point source pollution, and turbidity. In terms of utilities with *groundwater sources*, only 81% (127 of 156 who saw the question) identified one or more ways that their groundwater quality threats are triggered or worsened by weather events. Respondents with groundwater sources linked the following threats most often to weather events^3^ salinity (75%) and natural contaminants (74%). Closely behind these were agricultural land use contaminants, urban or industrial land use contaminants, and infrastructures. Further reporting of water quality impacts is in preparation.

### Preparedness dimensions

This section reports the results of three preparedness dimensions, which is followed by a summary of scores averaged by region.

#### Awareness

Statewide, all responses combined, 65% of respondents agreed at least somewhat that global climate is changing (151 out of 234) and slightly higher 68% agree that California’s climate is changing. Similarly, 65% agreed that climate change poses a risk to water quality globally, whereas a lower 53% of respondents agreed that climate change poses a threat to water quality *locally*.

Noteworthy is the large drop in respondents’ who *strongly agree* with the statements that climate change could threaten water quality globally versus locally (Figure S2). Of the 234 respondents, 84 (36%) *strongly agreed* that “climate change poses a risk to water quality globally”; whereas only 49 (21%) respondents *strongly agreed* that “climate change poses a risk to water quality locally.” In other words, this is a 42% drop in the degree of perceived local (vs. global) risk of climate change on water quality. Instead, the respondents selected a lower level of agreement (or stronger disagreement) with the statement that climate change poses a risk to water quality locally. The survey responses showed the respondents in coastal regions tended to agree more often than non-coastal regions that water quality is threatened locally by climate change.

#### Analytic capacity

We used three indicators to gauge analytic capacity: the presence of an operations model for water supply and its ability to incorporate climate variables, communication with climate experts, participation in an Urban Water Management Plan (which encourages the incorporation of climate change impacts review), and participation in an Integrated Regional Water Management Plan (IRWMP).

Of the 224 survey participants who responded to the question, 68 reported that they have an operations model. Of these 68 utilities with operations models, 35% (24) of these reported that the model as capable of integrating data from weather- and climate-related variables (temperature, precipitation). As expected, because of likely lower internal capacity, a lower percentage of the small utilities (1–10 employees) reported having operations model than those utilities with more employees (Supplemental Information, Figure S3).

#### Adaptation activity

Within the climate change planning section of the survey, we sought to find out what types of process-related adaptation/preparedness activities water utilities had participated in. We provided a list of common activities involved in an adaptation process, as consistent with Moser and Ekstrom (2010), and applied in case studies in Ekstrom and Moser (2014) and in surveys of Hart et al. (2012) and Shi et al. (2015). Nearly half of respondents (102/209) reported to have taken “no action” in adapting to climate change impacts on water quality. Of the respondents reporting no action, 75% (65 of the 87) reported in the survey that their system’s water quality problems presently are affected by extreme weather.

Over half of respondents (107/209) reported at least starting to become aware of the problems and need to prepare for climate change impacts on water quality.^4^ Just over 41% (86) of respondents reported having taken some action beyond having become aware of the issue, though the proportion of utilities reporting action varied widely by climate impact region.

To provide a preliminary test of what sorts of variables might enable the initiation of climate adaptation, we conducted a series of bivariate correlation tests with the advancement of climate adaptation activity against a series of system traits and responses (Supplementary Information, Table S12). Not surprisingly, the degree to which respondents reported to have water quality impacted by the drought positively correlated with their reported stage in climate adaptation. Higher perceived risk of climate impacts on local water quality also positively correlated with reported adaptation activity. Other variables that were positively correlated with advancement in adaptation processes included the amount of surface water used, provision of services beyond drinking water, involvement in regional processes, and communication with climate experts (Table S12).

In addition, those respondents at utilities where water quality threats worsen with extreme weather events are more often engaged in climate adaptation activities (as expected). Of those that are in utilities where weather affects water quality presently (154/183), 58% (89) reported to be actively involved in climate adaptation planning and/or management. In contrast, of those 29 utilities for which weather does not affect water quality, only 24% (9) report involvement in climate adaptation activities.

#### Summary climate preparedness by region

**Fig. 2:**
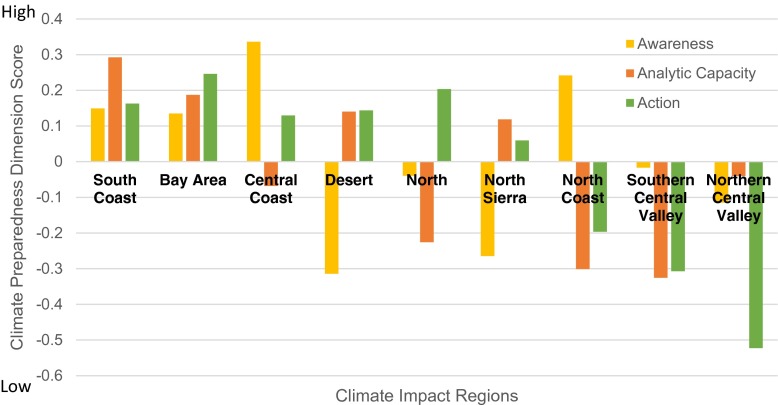
Summary of metrics gauging climate change awareness and adaptation activity in survey respondents. Results organized by climate impact region. Regions ordered by ranking of the Action score. The Southern Sierra was omitted from this summary to maintain anonymity (due to too few respondents)

Across the climate impact regions, two regions resulted in positive scores (where zero represents the mean) for all three dimensions: the Bay Area and South Coast (Fig. 2). Two regions also resulted in negative scores for all three dimensions: the Southern and Northern Central Valley (Fig. 2). While we expected analytic capacity scores to be more consistent across regions, these regions’ awareness scores were expected, based on prior knowledge. Awareness scores were consistent with a public opinion survey in which Central Valley residents expressed a lower perceived threat of climate change compared to the statewide average, whereas Los Angeles and the San Francisco Bay Area residents were more likely to express a higher perceived threat from climate change (Baldassare et al. 2015). Regional scores for adaptation activity also were consistent with observations of climate adaptation in the regional organizations in California that have emerged within the last decade. The three earliest groups were coastal (ARCCA 2016).

### Climate adaptation strategies

**Fig. 3:**
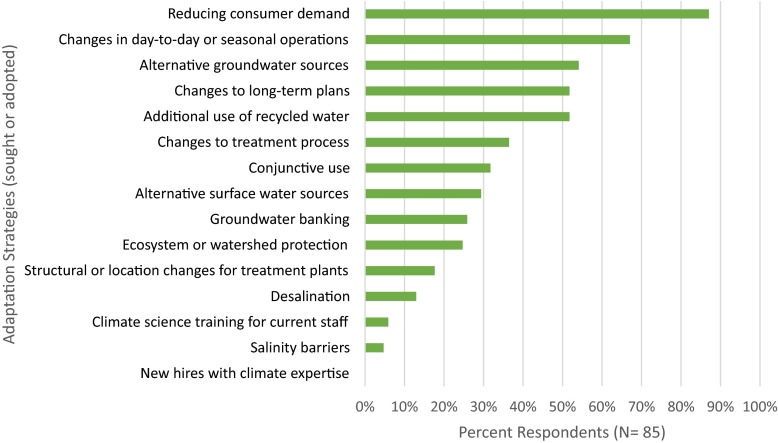
Adaptation strategies used by or discussed by respondent utilities for preparing for climate change impacts on water quality

Of the water utilities that reported having taken at least some adaptation-related action for climate change (selected activities listed within the Planning or Managing stages in the survey), the top reported adaptation strategy was reducing consumer demand (87%), which was mandatory statewide during the survey period (Fig. 3). The second most reported strategy was changes in short-term operations (67%), which was followed by using alternative groundwater sources (54%). The least reported strategies were those that could build internal organization capacity. No respondent reported the strategies of hiring new employees with climate change expertise, and only five reported having considered (or implemented) climate science training for staff.

### Information sources

Sixteen percent (34) of respondents reported that they communicate with climate experts. Those utilities that participate in IRWMP process, which includes a climate change planning standard, reported to have a slightly higher tendency to communicate with climate experts (11% of respondents without IRWMP communicate with climate experts compared to 20% of respondents with IRWMPs who do communicate with climate experts). Of those 34 that do communicate with climate experts, most experts (14/45) were reported to be in state government.

No matter the degree of awareness and degree of reported adaptation activity, trusted information sources most reported to be frequently used were state agencies (64%, 124 out of 195), followed by colleagues within the same utility (58%, 114). Respondents most often reported consulting academics and trade journals as “occasional” sources of information and data. More of the respondents who reported climate adaptation action also more frequently reported consulting professional conferences and academic scientists for data and information.

## Discussion

This section provides a synopsis of results with potential explanations, followed by next steps and recommendations. We found notable patterns of awareness, climate adaptation activity, and communication and the correlation of these with one another.

### Supply portfolio influence

Overall, we found there is a high awareness and concern that climate change will or is affecting water quality, but this is more so for those with surface water as at least part of their water portfolio. Respondent utilities relying on at least some surface water are farther along in all three dimensions of climate preparedness than those relying only on groundwater (Supplemental Information, Figure S4). This is not surprising, but still important to document, given that the climate impacts on surface water are better documented and modeled than those of groundwater (Taylor et al. 2012).

There are several plausible explanations for why utilities relying on only groundwater are not as far along in climate awareness and adaptation. Potentially contributing to differences in awareness scores, the connections between climate change and surface water are easier to see, monitor, model, and understand (Taylor et al. 2012) than groundwater. The IPCC (Jiménez Cisneros et al. 2014, WGII section 3.2.4) highlights the small number of studies of climate impacts on groundwater and climate change as an understudied area. Lack of information on how climate impacts groundwater availability and quality is also likely because fewer monitoring and treatment requirements historically have existed for groundwater systems compared to surface water.

Utilities have different monitoring, treatment and management expectations for groundwater. In 1992, the creation of a groundwater management plan was *encouraged* by the state (AB 3030), but not until passage of the 2014 Sustainable Groundwater Management Act (SGMA) were they mandated for certain basins. In contrast, there is a long tradition of surface water planning and management since the establishment of the California Water Code in 1943. Utilities with any source water from a surface water supply face higher monitoring and treatment requirements, relative to groundwater-only portfolios (EPA 1989; CA Safe Drinking Water Act 1996). And groundwater management happens at the basin or sub-basin, rather than utility, scale. And consistent with Conrad (2013), large suppliers, particularly those required to have an UWMP, are farther along.

Responses from utilities in coastal regions on average were the most advanced in terms of climate change awareness and adaptation action (Fig. 2). We explore three potential explanations. First, given that sea level rise has received wide media, research, and policy agenda attention for nearly the past decade, a concentrated effort has arisen along, and for the coasts, the awareness of this threat is more advanced generally than other threats of climate change (e.g., compared to public health, agriculture, and inland river flooding). Second, historically coastal utilities may face water scarcity issues with limited supply alternatives and tend not to rely as heavily on a state- or federal-centralized water distribution projects (with some exceptions). Third, the respondents answering on behalf of the largest utilities were also in coastal areas, so some of the higher scoring coastal regions may be more a reflection of the size of the utility than the geographic location. Larger utilities could be farther ahead than smaller-sized utilities because they may have more staff capacity to devote to climate change awareness and planning, among other reasons. The driver for why the coastal regions are farther ahead is not clear, but will be investigated through interviews with utility managers and staff to investigate this connection further.

### Adaptation strategies

Adaptation strategies have been classified in many different ways (Smit and Wandel 2006; Füssel 2007), and one increasingly popular evaluation is to classify the time horizon of change the strategy seeks to bring to the system—from reactionary, coping to transformative (Nelson 2009; O’Brien 2011; Haasnoot et al. 2013). The climate adaptation strategies respondents reported to be using or considering were dominated by a mix of coping and longer-term planning for alternative water supply sources. The majority of the alternative water supply sources were of groundwater (Fig. 3), which could have potentially long-term negative impacts in the state as groundwater is already being depleted in many areas (Famiglietti et al. 2011; Scanlon et al. 2012). Most notable—and perhaps concerning—is that very few of the respondents reported internal capacity building any of their adaptation efforts, and the most common strategy was coping mechanisms and those that could have long-term negative impacts. The most prevalent strategy reported is reducing consumer demand, which is a state-mandated short-term fix, and, therefore, could be considered a coping mechanism as opposed to longer-term capacity building and changes in organization and infrastructure that could support deeper transformative changes in the face of climate change.

### Next steps and recommendations

Past experience does not always drive awareness and adaptation (Hart et al. 2012), but based on our survey responses, it does more often than not for water quality management and climate change in California. Those respondents that reported having water quality affected by weather more often reported to be involved in climate adaptation activities. The challenge remains that the areas that could be the most severely affected by the current drought (Central Valley) are the regions with respondents that report the least adaptation activity and lowest analytic capacity. At the same time, the relatively strong correlation between the reported adaptation action and the perceived drought impacts warrants further investigation. Ongoing research focuses on drought impacts and responses in California among utilities to understand how the current drought has influenced utilities’ efforts in planning for climate change impacts. The current drought presents a suitable extreme event from which to understand how it has affected utility operations, whether it has affected decision-making, and when it has triggered or otherwise facilitated the incorporation of climate change projections in long-term planning.

This is the first evaluation of utility-level climate adaptation within water quality management at the local level in California. It is clear that the state takes climate change and adaptation seriously (Schwarzenegger 2008; DWR 2011; Moser et al. 2012; 379 SB 2015; CNRA et al. 2016); however, many local level efforts in small water systems largely are left to their own choice and devises on whether and how to adapt. Support is especially needed for smaller utilities, and gathering perspectives and experiences from these smaller utilities could reveal more precisely what they need (and how it may differ from larger utilities’ needs), as is planned in upcoming case studies.

The presented survey results can be useful to prioritize and develop a rudimentary typology of climate information needs across representative utilities. Based on our respondents, most groundwater-reliant systems are not yet adapting and the awareness and perceived risk is relatively low; therefore, translation- and application-oriented guidance of climate change information could match information needs for these types of utilities. The recent groundwater management legislation (2014 Sustainable Groundwater Management Act) presents a window of opportunity for including climate risks into long-term planning. Large surface water-reliant systems, which tended to be farther along in adaptation processes, could be upheld as examples for paving the path in adaptation, potentially applicable to other surface water utilities.

## Conclusion

Utilities are the lynchpin for adapting and securely maintaining drinking water in the face of climate change. State and federal level climate adaptation efforts are growing, but do not appear to be reaching utilities uniformly. This is likely the situation nationwide, where infrastructure needs updating and vulnerable populations often receive the brunt of impact from quality and economic cost increases. Anticipatory adaptation across governance levels needs to address and recognize the needs of managers of utilities of all different sizes and different portfolio types to safeguard the future of drinking water.

## Electronic supplementary material

Below is the link to the electronic supplementary material.

ESM 1 (DOCX 42 kb)

ESM 2 (DOCX 249 kb)

## Footnotes

1 UWMP contacts were used as backup when the point of contact from the SWRCB was duplicated for multiple water utilities. See the Supplementary Information for further details.

2 The SWRCB retains direct regulatory authority over water systems serving greater than or equal to 200 service connections; for systems with fewer than 200 service connections, regulatory authority is delegated to a local primary agency in 35 of California’s 58 counties.

3 Frequencies were divided by the total number of respondents who marked the issues as a threat to water quality.

4 For example, it means that when a respondent reported he is involved in assessing vulnerability and implementing adaptation strategies, we score that utility implementing adaptation strategies since this conveys a potentially more advanced stage of the adaptation process (Moser and Ekstrom 2010)

## References

[CR1] 379 SB (2015) California Senate Bill 379 Land use: general plan: safety element.

[CR2] American Association for Public Opinion Research (2015) Standard definitions final dispositions of case codes and outcome rates for surveys. 8th edition.

[CR3] Anderson J, Arora S, Ejeta M et al (2009) Using future climate projections to support water resources decision making in California. A Report from the California Climate Change Center, May 2009 CEC-500-2009-052-F. Available online: http://www.water.ca.gov/pubs/climate/using_future_climate_projections_to_support_water_resources_decision_making_in_california/usingfutureclimateprojtosuppwater_jun09_web.pdf

[CR4] ARCCA (2016) Alliance of Regional Collaboratives for Climate Adaptation (ARCCA) Initiatives (website) http://www.arccacalifornia.org/.Accessed 2016

[CR5] Archie KM, Dilling L, Milford JB, Pampel FC (2014). Unpacking the “information barrier”: comparing perspectives on information as a barrier to climate change adaptation in the interior mountain west. J Environ Manage.

[CR6] Baldassare M, Bonner D, Kordus D, and Lopes L (2015) Californians & the environment: PPIC statewide survey. A publication of the Public Policy Institute of California. http://www.ppic.org/content/pubs/survey/S_715MBS.pdf

[CR7] Bates BC, Kundzewicz ZW, Wu S, Palutikof JP (2008) Climate change and water. Technical paper of the Intergovernmental Panel on Climate Change. Geneva

[CR8] Bedsworth LW, Hanak E (2010). Adaptation to climate change. J Am Plan Assoc.

[CR9] Boehlert B, Strzepek KM, Chapra SC (2015). Climate change impacts and greenhouse gas mitigation effects on U.S. water quality. J Adv Model Earth Syst.

[CR10] Brown J (2015) Ca. Exec. Order B-29-15. (April 1, 2015), https://www.gov.ca.gov/docs/4.1.15_Executive_Order.pdf

[CR11] Cayan DR, Tyree M, Pierce D, Das T (2012) Climate and sea level change scenarios for California vulnerability and adaptation assessment. California Energy Commission. Publication number: CEC-500-2012-008

[CR12] Chen W-H, Haunschild K, Lund JR, Fleenor W (2010) Current and long-term effects of delta water quality on drinking water treatment costs from disinfection byproduct formation

[CR13] Christian-Smith J, Balazs C, Heberger M, Longley K (2013) Assessing water affordability: a pilot study in two regions of California. Oakland, CA

[CR14] CNRA, CDFA, CalEPA (2016) California water action plan 2016 update

[CR15] Conrad E (2012) Climate change and integrated regional water management in California: a preliminary assessment of regional approaches. Vol 4 in California Water Plan Update. A Report of the Department of Water Resources.

[CR16] Conrad E (2013) Preparing for new risks: addressing climate change in California’s urban water management plans. Sacramento, A Report to the Department of Water Resources. Available at http://www.water.ca.gov/climatechange/docs/UWMPClimateChangeReport_Final_June2013.pdf

[CR17] Delpla I, Jung AV, Baures E (2009). Impacts of climate change on surface water quality in relation to drinking water production. Environ Int.

[CR18] Department of Water Resources (2011) Climate change handbook for regional water planning. Sacramento

[CR19] Ekstrom JA, Moser SC (2014). Identifying and overcoming barriers in urban climate adaptation: case study findings from the San Francisco Bay area, California, USA. Urban Clim.

[CR20] Emelko MB, Silins U, Bladon KD, Stone M (2011). Implications of land disturbance on drinking water treatability in a changing climate: demonstrating the need for “source water supply and protection” strategies. Water Res.

[CR21] EPA (1974) Safe Drinking Water Act (SDWA). United States 42 U.S.C. § 300f

[CR22] Famiglietti JS, Lo M, Ho SL, et al (2011) Satellites measure recent rates of groundwater depletion in California’s Central Valley. Geophys Res Lett 38(3):L03811. doi:10.1029/2010GL046442

[CR23] Franco G, Cayan D, Moser SC, Hanemann, M.H. and M. A. Jones (2011). Second California Assessment: integrated climate change impacts assessment of natural and managed systems - an introduction. Clim Chang 109(Suppl. 1). doi:10.1007/s10584-011-0318-z

[CR24] Füssel HM (2007). Adaptation planning for climate change: concepts, assessment approaches, and key lessons. Sustain Sci.

[CR25] Haasnoot M, Kwakkel JH, Walker WE, ter Maat J (2013). Dynamic adaptive policy pathways: a method for crafting robust decisions for a deeply uncertain world. Glob Environ Chang.

[CR26] Hanak E, Gray B, Lund J et al. (2014) Paying for water in California. PPIC Publication. San Francisco, CA. http://www.ppic.org/content/pubs/report/R_314EHR.pdf

[CR27] Hart J, Grifman P, Moser SC et al (2012) Rising to the challenge: results of the 2011 Coastal California Adaptation Needs Assessment. USC Sea Grant Report. https://caseagrant.ucsd.edu/sites/default/files/CCSurveyReport_12MB.pdf

[CR28] Jiménez Cisneros BE, Oki T, Arnell NW, Benito G, Cogley JG, Döll P, Jiang T, Mwakalila SS (2014) Freshwater resources. In: Field CB, Barros VR, Dokken DJ, Mach KJ, Mastrandrea MD, Bilir TE, Chatterjee M, Ebi KL, Estrada YO, Genova RC, Girma B, Kissel ES, Levy AN, MacCracken S, Mastrandrea PR, White LL (eds) Climate Change 2014: Impacts, Adaptation, and Vulnerability. Part A: Global and Sectoral Aspects. Contribution of Working Group II to the Fifth Assessment Report of the Intergovernmental Panel on Climate Change. Cambridge University Press, Cambridge, pp 229–269. https://www.ipcc.ch/pdf/assessment-report/ar5/wg2/WGIIAR5-Chap3_FINAL.pdf

[CR29] Khan SJ, Deere D, Leusch FDL (2015). Extreme weather events: should drinking water quality management systems adapt to changing risk profiles?. Water Res.

[CR30] Kiem AS, Austin EK, Verdon-Kidd DC (2015) Water resource management in a variable and changing climate: hypothetical case study to explore decision making under uncertainty. J Water Clim Chang 7(2):jwc2015040. doi: 10.2166/wcc.2015.040

[CR31] Mastrandrea MD, Heller NE, Root TL, Schneider SH (2010). Bridging the gap: linking climate-impacts research with adaptation planning and management. Clim Change.

[CR32] Michalak AM (2016). Study role of climate change in extreme threats to water quality. Nature.

[CR33] Moser SC, Dilling L (2007) Creating a climate for change: communicating climate change and facilitating social change. Cambridge University Press, Cambridge

[CR34] Moser SC, Ekstrom JA (2010) A framework to diagnose barriers to climate change adaptation. Proc Natl Acad Sci. doi:10.1073/pnas.1007887107 10.1073/pnas.1007887107 PMC3009757 21135232

[CR35] Moser SC, Luers AL (2008). Managing climate risks in California: the need to engage resource managers for successful adaptation to change. Clim Change.

[CR36] Moser SC, Tribbia J (2007) More than information: what California’s coastal managers need to plan for climate change. National Center for Atmospheric Research, Sacramento, CA

[CR37] Moser SC, Ekstrom JA, Franco G (2012) Our Changing Climate 2012: Vulnerability & Adaptation to the Increasing Risks from Climate Change in California. California Energy Commission, Sacramento, CA. http://www.energy.ca.gov/2012publications/CEC-500-2012-007/CEC-500-2012-007.pdf

[CR38] Nelson DR, Adger WN, Lorenonzi I, O’Brien K (2009). Conclusions: transforming the world. Adapting to climate change: thresholds, values, governance.

[CR39] O’Brien K (2011). Global environmental change II: from adaptation to deliberate transformation. Prog Hum Geogr.

[CR40] Pal I, Towler E, Livneh B (2015). How can we better understand low river flows as climate changes?. Eos (Washington DC).

[CR41] Rayner S, Lach D, Ingram H, Houck M (2005). Weather forecasts are for wimps: why water resource managers do not use climate forecasts. Clim Change.

[CR42] Romero-Lankao P, et al. (2014) Climate change 2014: impacts, adaptation, and vulnerability. Part B. Regional aspects. In: Barros VR, Field CB, Dokken DJ, Mastrandrea MD, Mach KJ, Bilir TE, Chatterjee M, Ebi KL, Estrada YO, Genova RC, Girma B, Kissel ES, Levy AN, MacCracken S, Mastrandrea PR WL (ed) Contribution of Working Group II to the Fifth Assessment Report of the Intergovernmental Panel on Climate Change. Cambridge, UK, New York

[CR43] Scanlon BR, Faunt CC, Longuevergne L (2012). Groundwater depletion and sustainability of irrigation in the US High Plains and Central Valley. Proc Natl Acad Sci U S A.

[CR44] Schwarzenegger A (2008) Governor’s Executive Order # S-13-08

[CR45] Shi L, Chu E, Debats J (2015). Explaining progress in climate adaptation planning across 156 U.S. municipalities. J Am Plan Assoc.

[CR46] Smit B, Wandel J (2006). Adaptation, adaptive capacity and vulnerability. Glob Environ Chang.

[CR47] SWRCB (2015) Safe drinking water plan for California: report to the legislature. Sacramento

[CR48] Taylor RG, Scanlon B, Döll P (2012). Ground water and climate change. Nat Clim Chang.

[CR49] Westerling AL, Bryant BP (2008) Climate change and wildfire in California. Clim Change 87(Suppl:S231–S249). doi:10.1007/s10584-007-9363-z

