# Supplementary material for: Gauging climate preparedness to inform adaptation needs: local level adaptation in drinking water quality in CA, USA
Source: Clim Change. 2016 Dec 23;140(3):467–81. doi: 10.1007/s10584-016-1870-3 (PMC5266779; doi:10.1007/s10584-016-1870-3)
Supplement: Supplementary file 1 — (DOCX 42 kb) [file 10584_2016_1870_MOESM1_ESM.docx]

Please answer the survey questions based on your work experience with and knowledge of the **North Lakeport County Service Area #21.**

1. **What most closely describes your position at the water utility?**

- Director (1)
- General Manager (2)
- Operator (3)
- Water Quality Specialist (9)
- Engineer (6)
- Consultant (7)
- Planner (4)
- Finance Manager (5)
- Other (specify) (8) ____________________

1. **How long have you worked in water management?**

- Less than one year (1)
- 1-2 years (2)
- 3-5 years (3)
- 6-10 years (4)
- More than 10 years (5)

1. **What type of management decisions do you make in your current role?(Check all that apply)**

- Daily operations (1)
- Seasonal operations (2)
- Annual decision making (3)
- Long term planning: 5-10 years (4)
- Long term planning: beyond 10 years (5)
- Other (6) ____________________

***We would like to get more background about your utility.***

1. **How many employees work at your utility?**

- 1-10
- 11-50
- 51-100
- 101-250
- 251-500
- More than 500
- I don't know.

1. **Choose your utility size, based on population served:**

- Very Small (25-500 people)
- Small (501-3,300 people)
- Medium (3,301-10,000 people)
- Large (10,001-100,000 people)
- Very Large (100,001+ people)

1. **Based on our records, you purchase or receive at least some of your water supply from another water utility. What are the sources of this purchased supply? Select all that apply.**

- Surface water
- Groundwater
- Recycled water
- Unknown

1. **Does your water utility treat the water purchased (or otherwise received)?**

- Yes, all purchased water.
- Yes, some purchased water.
- No, do not treat purchased water.
- Other ____________________

1. **Other than chlorination, does your utility treat the surface water produced from intakes?**

- Yes, all of it.
- Yes, some of it.
- No.
- Not applicable.

1. **In addition to providing drinking water, does your utility provide any of the following other services? Select all that apply.**

- Electricity production
- Stormwater management
- Wastewater management
- Flood control
- Groundwater recharge and management
- Threatened or endangered species protection
- Other (specify) ____________________

***Now, we would like to better understand threats to water quality in your district.***

1. **Based on your experience, what is your utility's largest threat to water quality for its drinking water supplies?**
2. **In considering your district's surface water sources (used for drinking water), indicate how threatening each issue is to water quality.**

|  | Extremely serious threat | Very serious threat | Somewhat serious threat | Slightly serious threat | Not a threat | Unknown |
| --- | --- | --- | --- | --- | --- | --- |
| Point source pollution |  |  |  |  |  |  |
| Non-point source pollution |  |  |  |  |  |  |
| Turbidity |  |  |  |  |  |  |
| Eutrophication, algal blooms or low dissolved oxygen |  |  |  |  |  |  |
| Salinity |  |  |  |  |  |  |
| Infrastructure impairment or failure |  |  |  |  |  |  |
| Other _____ |  |  |  |  |  |  |
| Other ____ |  |  |  |  |  |  |

1. **Please indicate which weather events or environmental hazards worsen or trigger water quality issues.**

|  | Weather Events and Environmental Hazards | | | | | | | | | |
| --- | --- | --- | --- | --- | --- | --- | --- | --- | --- | --- |
|  | Drought | Low flows | Extreme storms, high flows | Landslides | Salt water intrusion | Increasing avg. temperatures | High temperatures | Wildfires | Other ______ | None |
| Point source pollution |  |  |  |  |  |  |  |  |  |  |
| Non-point source pollution |  |  |  |  |  |  |  |  |  |  |
| Turbidity |  |  |  |  |  |  |  |  |  |  |
| Eutrophication, algal blooms or low dissolved oxygen |  |  |  |  |  |  |  |  |  |  |
| Salinity |  |  |  |  |  |  |  |  |  |  |
| Infrastructure impairment or failure |  |  |  |  |  |  |  |  |  |  |
| Other __________ |  |  |  |  |  |  |  |  |  |  |
| Other _____ |  |  |  |  |  |  |  |  |  |  |

1. **If you selected "Other" weather events or environmental hazard that threatens water quality in your water system. Please briefly describe below.**

***As you are likely aware, California is experiencing a drought. We would like to ask a few questions on how the current drought is affecting water quality.***

1. To what extent has the current drought impacted your utility's water quality? Select the statement that most accurately reflects your experience.

- Very seriously impacted water quality
- Seriously impacted water quality
- Somewhat seriously impacted water quality
- Slightly impacted water quality
- Not impacted water quality
- Unknown

1. **If at all, please briefly describe how the drought has impacted your utility's water quality.**
2. **Has your utility changed water management practices to respond to or prepare for the drought's impacts on water quality?**

- Yes
- Somewhat
- No

1. **What is the time horizon of these changes your utility has made to prepare for or respond to the drought? (Select all that apply)**

- Daily operations changes
- Seasonal operations changes
- Annual planning and management changers
- Near term planning adjustments (>1-5 years)
- Long term plan revision (>10 years)
- Other ____________________

1. **Please briefly describe the changes in water management practices your utility has made due to the drought's impact on water quality.**


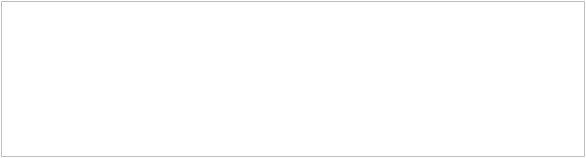


1. **Does your water utility have a written water shortage contingency or drought preparedness plan?**

- Yes
- No

1. **Does the contingency or preparedness plan include managing for water quality impacts?**

- Yes
- Somewhat
- No

1. **Has your utility implemented the plan due to the current drought?**

- Yes
- Somewhat
- No

1. **Whether or not your water shortage contingency or drought preparedness plan covers water quality, have you found it to be sufficient for managing water quality impacts of the drought?**

- Yes
- Somewhat
- No

1. **Have you found your utility's water shortage contingency or drought preparedness plan to be sufficient for managing water supply impacts?**

- Yes
- Somewhat
- No

***Finally, we would like to ask you some questions about climate change, its impacts on water quality, and planning at your water utility.***

1. **Please indicate your level of agreement with the following statements.**

|  | Strongly Agree | Agree somewhat | Neither Agree nor Disagree | Disagree somewhat | Strongly Disagree | I don’t know |
| --- | --- | --- | --- | --- | --- | --- |
| The global climate is changing. |  |  |  |  |  |  |
| California's climate is changing. |  |  |  |  |  |  |
| Climate change poses risks to water quality globally. |  |  |  |  |  |  |
| Climate change poses risks to water quality locally, for my water utility’s supply. |  |  |  |  |  |  |

1. **Please indicate which of the following climate change impacts are expected to threaten your utility's drinking water supply and its management in the next 50 years? (Select all climate impacts that apply).**

- Increase in frequency and/or severity in extreme storms, high flows
- Reduction in annual precipitation
- Shift in timing and seasonality of snow melt
- Increase in frequency and/or length of droughts
- Sea level rise
- Increase in frequency, severity and duration of high temperature events
- Shift in seasonal temperatures (e.g. earlier warming in spring)
- Increase in average temperatures
- Increase in frequency, severity and extent of wildfires
- Increase in water demand
- Other (fill in) ____________________
- None

1. **Which of the following climate change impacts do you expect to worsen water quality issues for your utility's surface water in the next 50 years? (Select all that apply).**

|  | **Water Quality Issues** | | | | | | | |
| --- | --- | --- | --- | --- | --- | --- | --- | --- |
|  | Point source pollution | Non-point source pollution | Turbidity | Eutrophication, algal blooms or low dissolved oxygen | Salinity | Infrastructure impairment or failure | Other Issue | *No water quality impact expected* |
| Increase in frequency and/or severity in extreme storms, high flows |  |  |  |  |  |  |  |  |
| Reduction in annual precipitation |  |  |  |  |  |  |  |  |
| Shift in timing and seasonality of snow melt |  |  |  |  |  |  |  |  |
| Increase in frequency and/or length of droughts |  |  |  |  |  |  |  |  |
| Sea level rise |  |  |  |  |  |  |  |  |
| Increase in frequency, severity and duration of high temperature events |  |  |  |  |  |  |  |  |
| Shift in seasonal temperatures (e.g. earlier warming in spring |  |  |  |  |  |  |  |  |
| Increase in average temperatures |  |  |  |  |  |  |  |  |
| Increase in frequency, severity and extent of wildfires |  |  |  |  |  |  |  |  |
| Increase in water demand |  |  |  |  |  |  |  |  |
| Other _________ |  |  |  |  |  |  |  |  |

1. **If in the previous question you selected "Other Issue" that you expect to worsen water quality for your utility's surface water in the next 50 years, please briefly describe this issue below.**
2. **In terms of preparing for climate change impacts on water quality, select all of the statements below which best describe your utility's activities to date:**

Understanding

- We are just beginning to become aware of the issue.
- We have started to gather some information to better understand the issue.
- We have completed an assessment on the issue.

Planning

- We are brainstorming a range of options to prepare for and manage climate change risks.
- We have completed an assessment of potential response options.
- We have selected a subset of response options to move forward with.

Managing

- We have begun implementing the selected response options.
- We are monitoring how well the implemented responses are working out.
- We are evaluating and reassessing how well the implemented options are faring.

No action

- We have not looked at all into preparing or planning for the impacts of climate change on water quality.

1. **If any, what strategies are you considering or implementing to manage for climate change impacts on water quality?**

- Alternative surface water sources
- Alternative groundwater sources
- Desalination
- Conjunctive use
- Ecosystem or watershed protection
- Groundwater banking
- Additional use of recycled water
- Reducing consumer demand
- Structural or location changes for treatment plants
- Salinity barriers
- Changes to treatment process
- Changes in day-to-day or seasonal operations
- Changes to long-term plans
- New hires with climate expertise
- Climate science training for current staff
- Other (fill in) ______________________________________

1. **Does your water utility participate in any of the following local and regional processes?**

- Integrated Regional Water Management Plan
- Regional Flood Management Plan
- Regional Climate Adaptation Collaborative
- Groundwater Sustainability Agency Formation / Groundwater Sustainability Plans (SGMA)
- Local Hazard Mitigation
- City or County General
- Other (list here) ____________________
- None

1. **To what degree do you consult the following sources to obtain data and information you need for your work related to managing drinking water quality?**

|  | All the time | Frequently | Occasionally | Rarely | Do not use in my work |
| --- | --- | --- | --- | --- | --- |
| Websites |  |  |  |  |  |
| Social Media |  |  |  |  |  |
| Colleagues at work |  |  |  |  |  |
| State Agencies |  |  |  |  |  |
| Federal agencies |  |  |  |  |  |
| Consultants |  |  |  |  |  |
| Colleagues in other water systems |  |  |  |  |  |
| Experts at research/academic institution(s) |  |  |  |  |  |
| Trade journals (scientific or other professional) |  |  |  |  |  |
| Professional listservs |  |  |  |  |  |
| Non-governmental organizations (NGOs) |  |  |  |  |  |
| Professional conferences or meetings |  |  |  |  |  |
| Other ___ |  |  |  |  |  |

1. **Does your utility have an operations model you use to estimate water deliveries or that is used for future water delivery planning purposes?**

- Yes (specify model below) ____________________
- No

1. **If so, is this operations model capable of integrating data from variables such as temperature, precipitation, streamflow, evapotranspiration, or groundwater recharge rates?**

- Yes (specify how) ____________________
- No

1. **Do you communicate with any climate experts? Check all that apply and please specify which organizations in the space provided.**

- No.
- Yes, in my agency.
- Yes, at other water utilities. ____________________
- Yes, at a university or other research institute. ____________________
- Yes, at the State government. ____________________
- Yes, at the Federal government. ____________________
- Other ____________________

1. **Which of the following would be useful to your utility to manage climate change impacts on water quality? Select all that apply.**

- Local or regional-relevant monitoring data
- Improved forecasts or projections
- Tools/dashboards to help find data available
- Having relevant science translated
- Other ____________________
- None

***Thank you for taking the time to fill out this survey. We appreciate your feedback! Your responses help provide a better idea of threats to water quality across California water utilities and what managers need to safeguard this valuable asset.***

**Please indicate below if we can follow up with you to gather more information on your responses.**

- Yes (provide your name and email or phone number) ____________________
- No
