# Supplementary material for: Gauging climate preparedness to inform adaptation needs: local level adaptation in drinking water quality in CA, USA
Source: Clim Change. 2016 Dec 23;140(3):467–81. doi: 10.1007/s10584-016-1870-3 (PMC5266779; doi:10.1007/s10584-016-1870-3)
Supplement: Supplementary file 2 — (DOCX 249 kb) [file 10584_2016_1870_MOESM2_ESM.docx]

# Supplemental Information

This supplementary information provides two sections of further details to complement the main manuscript: 1. a survey disposition report containing details of the survey development and complex sampling, along with response rates and sampling bias; and 2. additional description of the index development and further examination of the survey results.

# 1. Disposition Report

1.1 Study Overview

The California Water Quality and Extreme Events Survey was an online survey distributed to 756 drinking water utilities in California that met certain criteria. The term “utility” here captures special districts, local agencies, retailers, wholesalers, municipalities and other organizations that supply drinking water in California. Drinking water utilities manage one or more public water system (PWS) in California. Utilities were eligible for the survey if they had first, submitted their annual compliance report to the SWRCB State Water Resources Control Board (SWRCB) drinking water division’s (DDW) 2014 annual reporting process and second, reported more than 200 Potable Service Connections (SC). For systems with fewer than 200 service connections, regulatory authority is delegated to a Local Primary Agency in 30 of California’s 58 counties. Thirty of California’s 58 counties are considered to be a Local Primacy Agency (LPA), meaning that are delegated to regulate PWS that serve less than 200 SCs; these counties regulate statewide approximately 3,700 small PWS (SWRCB 2014). The SWRCB DDW directly regulates any small PWS not delegated to an LPA county and PWS serving 200 or more SC for all of California.

Survey participants are the named contact person for a public water systems and have diverse roles in managing the drinking water provided by these systems from general manager to water quality engineer. They were named through their participation in submitting their system’s data to the SWRCB DDW’s 2014 annual compliance report. We also received contact details for the person that submitted a 2010 Urban Water Management Plan (UWMP) to the Department of Water Resources on behalf a single or multiple PWS. In several instances, UWMP incorporate more than one PWS.

### Survey Purpose and Goals

Many previous studies have explored climate change impacts on water supply and scarcity, but few have delved deeply into the intersection of climate change and water quality. Drinking water systems have critically important functions in mediating the severity of climate impacts on the communities they serve, including ensuring the provision of safe, drinking water in the face of potential impacts. The survey asked participants about their experience in dealing with water-quality issues and perception of threats to quality due to extreme climate and weather events like droughts, floods etc.. The purpose of the study was to gather information from water resource managers on considerations of past and future risks of extreme events on drinking water quality management and to identify the type of information they need to manage those risks. There will be a parallel study with air quality managers. We also sought to collect information about utilities’ experiences, progress/awareness in adaptation and current threats – to inform future case study selection.

### The Questionnaire

The online questionnaire was developed in Qualtrics (license purchased through UC Davis) by Julia Ekstrom and Louise Bedsworth, with support from a Graduate Student Researcher, Amanda Fencl and suggestions by UC Davis faculty Mark Lubell and Gwen Arnold, and managers at the Department of Water Resources. The structure and some of the questions of the web questionnaire were informed by a similar survey used in the 2012 by Julia Ekstrom, as part of the California Coastal Adaptation Needs Assessment (Finzi-Hart et al., 2012), which was based on Tribbia and Moser (2008).

1.2 Characteristics of the Sample

We sought to construct a contact list to invite utilities in California that provide residential drinking water. To construct the contact list, we used contacts from two different state agencies: (1) State Water Resource Control Board (SWRCB) Annual Drinking Water Survey for the year 2014 (the most recent at time of the survey); and (2) Department of Water Resources Urban Water Management Plan 2010 submissions. Many points of contact in the SWRCB drinking water survey list were repeated for multiple water systems. Given that we wanted to send each person an invitation to only one survey, we cross-checked the Department of Water Resources’ list of contacts to find an alternative contact person for each of those water systems that had duplicate contacts from SWRCB and replaced them with the alternative when it existed. As described in detail below, for the final list (756 people), we only kept those systems with a unique point of contact and those with supply source portfolio data (groundwater, surface water or purchased) in their 2014 submitted annual report.

A public water system is defined as a water system serving 15 or more service connections, or 25 or more users for 60-plus days per year. Public water systems are divided into three principle classifications by the transience of the population they serve: community water systems (CWS), non-transient non-community water systems (NTNC), and transient, and non-community water systems (TNC). All three of these system types submit Annual Compliance Report data to the Division of Drinking Water. **To be included as a target water utility, contacts had to meet Criteria 1 and Criteria 2.**

The sampling method, response rates and results presented below frequently are reported by climate impact region (Figure S1, CEMA & CNRA 2014).


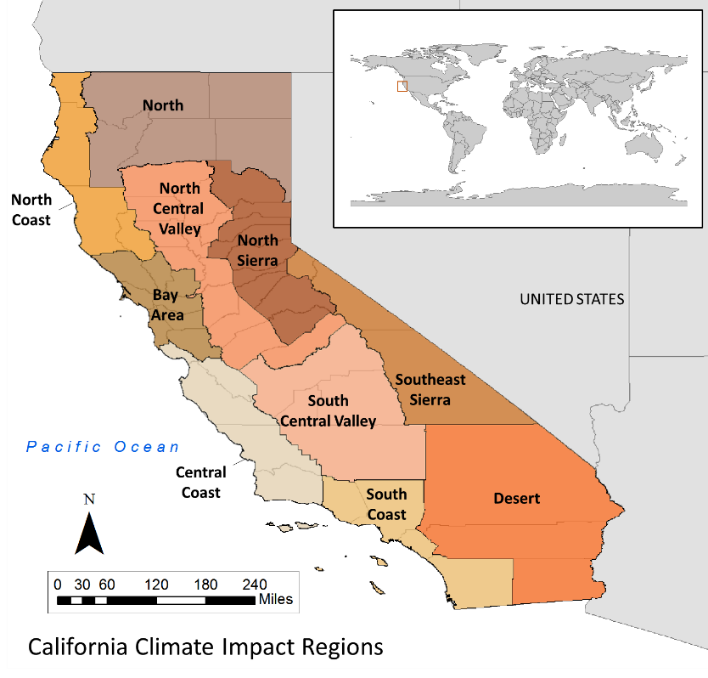


Figure S1. Map of California Climate Impact Regions, as defined in CEMA and CNRA (2012)

### CRITERIA 1: Eligible Public Water System

Criterion 1A. Must be a public water system (district, agency, utility etc.) in California that provides drinking water (N= 8302).

Table S1**.** Public Water System (PWS) counts, service connections and population served totals by climate impact region in California^[[1]](#footnote-1)^.

| **Climate Impact Region** | **PWS Count** | **Sum of Service Connections** | **Sum of Population Served** |
| --- | --- | --- | --- |
| Bay Area | 1137 | 1,915,997 | 7,591,354 |
| Central Coast | 899 | 410,057 | 1,497,104 |
| Desert | 781 | 1,296,669 | 4,557,587 |
| North | 370 | 83,698 | 300,915 |
| North Coast | 340 | 96,966 | 385,746 |
| North Sierra | 985 | 298,254 | 942,576 |
| Northern Central Valley | 1642 | 1,142,976 | 4,148,643 |
| South Coast | 709 | 3,761,602 | 20,733,263 |
| Southeast Sierra | 273 | 15,526 | 55,005 |
| Southern Central Valley | 1144 | 615,721 | 2,521,720 |
| Unknown/ Missing Information | 22 | 35 | 885 |
| **Total** | **8,302** | **9,637,501** | **42,734,798*** |

*** “**Sum of Population Served” is greater than the current population of California because wholesalers reported populations are often in duplication of retailers’ reported population and counts include water suppliers to non-residences (i.e. gas stations, schools, restaurants, and highway rest stops) in addition to residential connections.

Criterion 1B. Public water systems must have greater than or equal to 200 potable service connections (2,915). We focused on these larger systems for two reasons. First, within 35 of the state’s 58 counties, drinking water management for the smallest systems (with <200 service connections) is overseen by individual county offices, rather than by the SWRCB. Second, the majority are non-community or non-public water systems, whereas this project sought to gather input from those purveyors to households, which are largely community water systems (Table S2).

Table S2**.** Number of water systems by type and number of service connections. Source: SDWIS download 4/2016 (DWP 2016)

|  | Public Water Systems in California | | |
| --- | --- | --- | --- |
|  | **<200 service connections** | **≥200 service connections** | **All** |
| Community | 1350 | 1650 | 3000 |
| Non-Community (Transient) | 2460 | 796 | 3256 |
| Non-Public | 452 | 2 | 454 |
| Non-Transient Non-Community | 1036 | 467 | 1503 |
| Unknown | 89 | 0 | 89 |
| **Total** | **5387** | **2915** | **8302** |

Table S3. Shows the universe of systems in California by region and size that have ≥200 service connections, which meet the system size criteria for the survey.

|  | Number of systems with ≥200 service connections by population served in California | | | | | |
| --- | --- | --- | --- | --- | --- | --- |
|  | **Very Large (>100,000 people)** | **Large (10,001-100,000 people)** | **Medium (3,301-10,000 people)** | **Small (501-3,300 people)** | **Very Small (<501 people)** | **Total System Count** |
| Bay Area | 16 | 50 | 20 | 72 | 173 | 331 |
| Central Coast | 2 | 27 | 27 | 84 | 112 | 252 |
| Desert | 12 | 46 | 34 | 105 | 119 | 316 |
| North | 0 | 5 | 10 | 41 | 76 | 132 |
| North Coast | 0 | 8 | 12 | 54 | 84 | 158 |
| North Sierra | 2 | 11 | 27 | 111 | 193 | 344 |
| Northern Central Valley | 10 | 48 | 48 | 140 | 265 | 511 |
| South Coast | 43 | 117 | 45 | 86 | 118 | 409 |
| Southeast Sierra | 0 | 0 | 2 | 17 | 39 | 58 |
| Southern Central Valley | 5 | 37 | 30 | 122 | 210 | 404 |
| **Total** | **90** | **349** | **255** | **832** | **1389** | **2915** |

CRITERIA 2. AVAILABLE CONTACT INFORMATION AND SUPPLY SOURCE

For the second set of criteria, we targeted systems will available water supply source data, which we used in the back-end of the survey design to instruct the branched logic of which questions were viewed by each respondent. The SWRCB Annual Drinking Water Survey 2014 collected this information, along with email addresses for most of the participating public water system. We used this list as our primary set of eligible systems, creating a point of contact list. We filtered this list for a unique set of email addresses (Points of Contact, POCs). In many cases, the same person was listed as the contact person for multiple utilities. In these situations, we checked the DWR-provided list of Urban Water Management Plan 2010 contacts for an alternative contact person for each of the water systems that had duplicate contacts from SWRCB and replaced them with alternatives when available.

The final contact email list constructed was composed of 756 people, which represented 925 public water systems. Of the final contact list, 363 came from the SWRCB annual survey and did not have an Urban Water Management Plan. The remaining 393 point of contacts came from the DWR Urban Water Management Plan contact list. Many Urban Water Management Plans are written on behalf of multiple public water systems that are adjacent or otherwise connected to one another and overseen by the same utility. Therefore, in many cases these contacts represented more than one public water system, which is why the number of systems represented overall is higher than actual contacts invited to participate in the survey.

Table S4. Final break-down of public water systems (PWS) covered by the invited point of contacts by climate impact region (% out of total eligible systems with ≥200 service connections)

| **Climate Impact Region** | **Count of PWS (% of total)** | **Sum of Potable Service Connections (% of total)** |
| --- | --- | --- |
| Bay Area | 93 (28) | 1,628,570 (85) |
| Central Coast | 77 (31) | 355,208 (89) |
| Desert | 113 (36) | 1,125,421 (87) |
| North | 22 (17) | 28,519 (36) |
| North Coast | 54 (34) | 77,415 (84) |
| North Sierra | 109 (32) | 222,880 (78) |
| Northern Central Valley | 125 (24) | 829,323 (74) |
| South Coast | 231 (56) | 3,373,115 (90) |
| Southeast Sierra | 11 (19) | 7554 (69) |
| Southern Central Valley | 90 (22) | 456,974 (76) |
| **Total** | **925 (32)** | **8,104,979 (85)** |

Web Survey Contact Procedures

The Policy Institute sent an email invitation to those with email addresses included in the original contact list on July 29, 2015. The message signatory was Julia Ekstrom, and each email included a summary of the project and the link to access their utility’s unique online survey. Instructions for opting-out of accessing the survey were also included at the end of the email message in the event the recipient was uninterested in participating and receiving future correspondence.

The Policy Institute sent five additional, weekly email messages to the original sample list during the fielding period (see table below). All messages were similar in that the signatory was consistent and contained a link to the survey. However, these messages were not sent to anyone who had already responded, refused to participate by opting out at the time a message was sent. A final reminder message on September 1^st^, 2015, alerted the recipients that the study was closing soon and this was their last opportunity to participate.

Information Regarding Sources of Survey Error and Sampling Bias

Surveys of this kind are sometimes subject to types of inaccuracies for which precise estimates cannot be calculated. For example, findings may be influenced by events that take place while the survey is in the field, like the current drought, recent wildfires, the new state groundwater management requirements, or the local political opinion of climate change. Events occurring since the time the surveys were completed could have changed the opinions reported here. Sometimes questions are inadvertently biased or misleading. Additionally, the views of people who responded to the survey may not necessarily replicate the views of those who refused to fill out their questionnaires, or the views of other respondents at the same establishment.

Sampling bias tends to be more present in surveys that use convenience sampling, as opposed to those that employ random sampling. Because we relied on a small set of eligibility criteria and then relied on availability of contact information, our method is a combination of purposeful and convenience sampling. For this reason, we present the response rates reporting both the respondents (n) compared to the invited point of contacts (N), and also compared to the universe of water utilities eligible (U) for the study (Table S8 and Table S10).

Final Disposition Summary

The following table classifies every case according to its final disposition. These dispositions are initially based on the guidelines for final disposition codes established by the American Association for Public Opinion Research (AAPOR) Standard Definitions for Final Dispositions of Case Codes, 2015. Further explanation of the disposition codes can be found in Appendix A.

| **Disposition Codes** | **Panel A Contacts PWSs^1^** | **Panel B**  **Contacts with UWMPs** | **Total** | **Code** |
| --- | --- | --- | --- | --- |
| **Completion (1.1)** | 115 | 103 | 218 | I |
| **Partial completion (1.2)** | 20 | 21 | 41 | P |
| **Completion Total** | 135 | 124 | 259 |  |
| **Explicit refusal (2.11) [opt-out] ^2.^** | 9 | 3 | 12 | R |
| **Implicit refusal (2.12) [break-off]** | 8 | 24 | 32 | R |
| **Non-contacts (2.20) [no response]** | 209 | 240 | 449 | R |
| **Refusal and break-off Total** |  |  |  | R |
| **Email returned/Undeliverable (3.30)** | 2 | 2 | 4 | UO |
| **Total** | **363** | **393** | **756** |  |
| Notes: ^1.^ Panel A PWS have no UWMP because they do not meet the DWR minimum threshold of water volume or population served; ^2.^ 15 opted out by unsubscribing from emails, but 3 are included in 1.2 or 1.1 because they completed a sufficient amount of the survey before unsubscribing | | | | |

1.3. Response Rates Summary

Using the above disposition codes, the response, cooperation and refusal rates for each Panel, A and B, and for the entire survey as follows and were calculated using the AAPOR’s calculator^[[2]](#footnote-2)^:

- **Response Rates (RR)** calculation include both completed (RR1) and completed and partial responses together (RR2). **RR1** is the minimum response rate—the number of completed surveys divided by the total number of surveys (complete plus partial) plus the number of non-responses (refusal and break-off etc.) plus all cases of unknown eligibility (UO). **RR2** is calculated similarly to RRI, but also includes partial responses.
- **Cooperation Rates (COOP)** are also calculated for the proportion of all cases surveyed or contacted of all eligible units and refusals from known respondents. **COOP1** is the minimum cooperation rate, is the number of complete surveys divided by the number of surveys (complete plus partial) plus the number of non-responses that involve the identification of and contact with an eligible respondent (refusal and break-off plus other). Similar to RR2, **COOP2** also includes partial responses.
- **Refusal Rate** (REF1): A refusal rate is the proportion of all cases in which a respondent refuses to do complete the survey, or breaks-off the survey with insufficient responses to be considered a partial response. **REF1** is the number of explicit and implicit refusals divided by the surveys (complete and partial) plus the non-respondents (refusals, non-contacts, and others) and cases of unknown eligibility (UH + UO).

Table S5. Overall Response Rate

| **AAPOR Rate Type:** | **Panel A** | **Panel B** | **Total** | **Equation*** |
| --- | --- | --- | --- | --- |
| **RR1** | 0.317 | 0262 | 0.288 | I / (I + P) + (R + NC) + (UO) |
| **RR2** (inc. partial responses) | 0.372 | 0.316 | **0.343** | (I + P) / (I + P) + (R + NC) + (UO) |
| **COOP1** | 0.757 | 0.682 | 0.719 | I / (I+P+R) |
| **COOP2** (inc. partial responses) | 0.888 | 0.821 | **0.855** | (I+P)/(I+P+R) |
| **REF1** | 0.047 | 0.069 | 0.058 | R/((I+P)+(R+NC+O) + UO)) |
| *I = Complete interview (1.1); P = Partial interview (1.2); R = Refusal and break-off (2.11, 2.12);  NC = Non-contact (2.20); UO = Email undeliverable (3.30). | | | | |

In summary, the overall survey Response Rate 2 is 34.3% and Cooperation Rate 2 is 84.6% for the 2015 California Water Quality and Extreme Events Survey. Response rates were slightly higher for Panel A (respondents without Urban Water Management Plans) compared to those in Panel B (respondents with UWMP).

## Response Rates and Representation of Eligible Universe by Water System Attributes

In this section we report response rates and proportions that the respondents represent the eligible universe of water systems using two units of analysis: counts of public water systems and the sum of service connections covered by the water systems. The counts of water systems include by geographic location (climate impact regions, Table S6-S7), water systems’ size class (Table S8), and water supply sources (Table S9). The coverage of service connections represented by the survey respondents is reported by climate impact region (Table S10).

### Responses Climate Impact Region

The number and rate of responses by counts of people invited and having responded to the survey are shown in Table S6. Because some respondents answered on behalf of multiple public water systems, Table S7 contains the counts and rates by water system that are represented in the invited contacts and respondents.

Table S6. Response Rate by Climate Impact (CI) region. Counts based on point of contacts.

| **Climate Impact Region** | **Response Count, n** | **Total Surveyed, N** | **Response Rate (n/N)** |
| --- | --- | --- | --- |
| Bay Area | 34 | 85 | 40% |
| Central Coast | 22 | 67 | 33% |
| Desert | 29 | 98 | 30% |
| North | 10 | 22 | 45% |
| North Coast | 14 | 39 | 36% |
| North Sierra | 25 | 67 | 37% |
| Northern Central Valley | 36 | 92 | 39% |
| South Coast | 54 | 190 | 28% |
| Southeast Sierra | 4 | 11 | 36% |
| Southern Central Valley | 31 | 85 | 36% |
| **Total Count** | **259** | **756** | **34%** |

Table S7. Response Rate by Climate Impact region. Counts based on public water systems represented in the point of contacts.

|  | **Eligible Universe of PWS (U)** | **#PWS in Invited Contacts (N)** | **Responses represented in Eligible Universe (N/U)** | **Respondent PWS (n)** | **Respondents representation in Eligible Universe (n/U)** | **Margin of Error at 95% confidence interval*** |
| --- | --- | --- | --- | --- | --- | --- |
| Bay Area | 331 | 93 | 28% | 38 | 11% | **±**15% |
| Central Coast | 252 | 77 | 31% | 27 | 11% | **±**18% |
| Desert | 316 | 113 | 36% | 37 | 12% | **±**15% |
| North | 132 | 22 | 17% | 11 | 8% | **±**28% |
| North Coast | 158 | 54 | 34% | 15 | 9% | **±**24% |
| North Sierra | 344 | 109 | 32% | 32 | 9% | **±**17% |
| Northern Central Valley | 511 | 125 | 24% | 48 | 9% | **±**13% |
| South Coast | 409 | 231 | 56% | 59 | 14% | **±**12% |
| Southeast Sierra | 58 | 11 | 19% | 4 | 7% | **±**48% |
| Southern Central Valley | 404 | 90 | 22% | 40 | 10% | **±**15% |
| **Total Count** | **2915** | **925** | **32%** | **311** | **11%** | **±5%** |

* Margin of error was calculated using U as the total population and public water systems (n) represented with the respondents as the sample.

### Response by Size Class (Population Served)

By size class the water systems represented in the survey respondent sample varied widely in terms of the proportions represented by size class (Table S8). The smallest size class of water systems proportionally participated the least in the survey (with only 3% of the eligible universe of public water systems is represented in the respondents). While this is not surprising given they have fewer staff and thus likely to have less time to fill out a voluntary survey, the discrepancy does highlight the limitations of generalizing the results of the study to the larger population of water systems.

Table S8. Public water systems (PWS) represented in the survey participants relative to the eligible universe of water systems in California, by population served categories. Eligible universe includes systems with ≥200 service connections.

| **Size Class by Population Served (ps)** | **Eligible Universe of PWS (U)** | **% of PWS by size class in Eligible Universe (U_ps_/U_t_)** | **# PWS represented in responses (n)** | **Responses represented in Eligible Universe (n_s_/U_s_)** | **% of respondents by size class (n_s_/n_t_)** | **Margin of Error at 95% confidence interval*** |
| --- | --- | --- | --- | --- | --- | --- |
| Very Small (<501) | 1,389 | 48% | 44 | 3% | 14% | **±**15% |
| Small (501-3,300 people) | 832 | 26% | 105 | 13% | 34% | **±**9% |
| Medium (3,301-10,000 people) | 255 | 9% | 48 | 19% | 15% | **±**13% |
| Large (10,001-100,000 people) | 349 | 12% | 94 | 27% | 30% | **±**9% |
| Very Large (>100,000 people) | 90 | 3% | 20 | 22% | 6% | **±**19% |
| **Total Count (t)** | **2,915 (U_t_)** | **100%** | **311 (n_t_)** | **11%** | **100%** | **±5%** |

* Margin of error was calculated using U as the total population and public water systems (n) represented with the respondents as the sample.

### Response by Source of Water Supply

In addition to varying by size and location, public water systems also vary in terms of the sources of their water. Table S9 shows the invited and respondent utilities broken down by where their water comes from. We use four categories, none of which are exclusive, meaning for example that systems were tallied under Any Groundwater and Any Purchased Water if they have their own wells and also purchase water from a wholesaler. Based on the water supplies, the proportions of the water systems that responded to the survey (n/s) closely reflected those that were invited to the survey (N/S).

Response rates were consistent across all water source types, ranging from 31% to 36%. The response rate here means that, for example, of the 574 utilities invited to the survey that use at least some groundwater, 33% participated in the survey. Specific water supply source data were only available for those water systems that reported volumes to the SWRCB 2014 annual drinking water report, therefore, the eligible universe is not included in Table S9.

Table S9. Responses, number of points of contact, respondents, and rate broken down by water supply source. Source data on water supply portfolio type from SWRCB 2014 Drinking Water Survey.

| **Water Supply Type (non-exclusive)** | **# Contacts Surveyed (N)** | **# Total Surveyed PWS (S, across all portfolio types)** | **Portion of total surveyed (N/S)** | **# Responses (n)** | **Proportion of respondents represented by water source (n/s*)** | **Response Rate (n/N)** |
| --- | --- | --- | --- | --- | --- | --- |
| Any Groundwater (direct intake) | 574 | 756 | 0.76 | 192 | 0.62 | 0.33 |
| Any Surface Water (direct intake) | 196 |  | 0.26 | 70 | 0.23 | 0.36 |
| Both Surface and Groundwater   (direct intake) | 97 |  | 0.13 | 30 | 0.10 | 0.31 |
| Any Purchased Water | 265 |  | 0.35 | 87 | 0.28 | 0.33 |

* *s* is the number of public water systems represented through respondents (s=311).

### Service Connections Response Rate

To understand the portions of service connections represented through the survey respondents, Table S10 presents the counts and proportions evaluated. We observe that 24% of the total service connections statewide (from eligible systems with ≥200 service connections) are represented through the survey respondents. This is to illustrate the coverage of the survey, but is not attributed to interpreting the survey responses.

Table S10. Survey response rate coverage based on service connections (SC) represented in responses.

|  | **Total Responses SC (n)** | **Total Surveyed SC (N)** | **Eligible Universe of SC (U)** | **Response Rate by SC (n/N)** | **SC represented in respondents (n/U)** |
| --- | --- | --- | --- | --- | --- |
| Bay Area | 486,537 | 1,628,570 | 1,905,872 | 0.30 | 0.26 |
| Central Coast | 122,113 | 355,208 | 400,645 | 0.34 | 0.30 |
| Desert | 459,706 | 1,125,421 | 1,288,056 | 0.41 | 0.36 |
| North | 13,480 | 28,519 | 78,707 | 0.47 | 0.17 |
| North Coast | 33,350 | 77,415 | 92,532 | 0.43 | 0.36 |
| North Sierra | 93,564 | 222,880 | 285,992 | 0.42 | 0.33 |
| Northern Central Valley | 266,993 | 829,323 | 1,127,881 | 0.32 | 0.24 |
| South Coast | 701,854 | 3,373,115 | 3,741,210 | 0.21 | 0.19 |
| Southeast Sierra | 1354 | 7554 | 10,875 | 0.18 | 0.12 |
| Southern Central Valley | 112,548 | 456,974 | 602,672 | 0.25 | 0.19 |
| **Grand Total** | **2,291,499** | **8,104,979** | **9,534,443** | **0.28** | **0.24** |

## Sample, Selection and Non-Response Bias

We sought to take a census of the water utilities with over 200 service connections across California. However, we were limited to the available contact lists, which directed our final sample. Therefore, we used a mixture of purposeful and convenience sampling methods, which can produce a sampling bias because the invited participants are not selected at random or to evenly represent characteristics of a population. We found that those utilities invited to participate in the survey were closely representative of the proportions seen in the universe of eligible water utilities. But due to a large portion of non-responses among utilities that serve the fewest people, a higher proportion of respondents participated in the survey that were very large (22%) and large (27%) compared to the very small water systems that serve 500 people or less (3%) (Table S8). This means that proportionally, the survey results present an over-representation of the largest water systems and proportionally under-representing the smallest systems. One likely reason for this pattern of non-response could be that the smaller systems typically do not have as much staff capacity as larger systems to respond to a voluntary survey.

Another possible bias relates to participation. Given that the title of the survey referred to extreme events and that it was distributed through a university email, these may have influenced those who did or did not respond. We expect that utilities more interested in climate change or generally in science would be more likely to respond, whereas those utilities with little interest in (or low regard for) climate change science or distrust of universities may be more likely to not participate. We would expect these qualities to positively correspond with low or no climate adaptation activity. Because of this, we expect that the respondents of the survey are more advanced that the non-response utilities in California. Because of these biases, we only cautiously generalize results to the total population of eligible utilities in California.

# Appendix A. Disposition Codes Applied in Results

Disposition code explanations as applied to the *California Water Quality and Extreme Events Survey*, based on the 2015 Standard Definitions from the American Association for Public Opinion Research (APPOR).

| Completion (1.1) |
| --- |
| Partial completion (1.2) |
| Explicit refusal (2.11) [opted-out] |
| Implicit refusal (2.12) |
| Non-contacts (2.20) |
| Email returned/Undeliverable (3.30) |

1.0 Returned questionnaire:

- 1. Complete. User completed a majority of the questions, and submitted their responses at the end.
  2. Partial or break-off with sufficient information, respondents had to have answered “Question 17: Based on your experience, what is your utility's largest threat to water quality for its drinking...“ or one other substantive question.

2.0 Eligible “non-interviews”:

*2.11 Explicit Refusal*

Represents those people who used the survey link to “Opt Out” explicitly.

*2.12 Implicit refusal / break-off or partial with insufficient information*

In the initial disposition table, this includes respondents who completed the first page but went no further, despite additional email invitations. They are considered refusals rather than a non-response because it is known that the email was received and an assumption is being made that the prospective respondent believes s/he is likely to be eligible.

In the adjusted disposition table, this includes the likely proportion of people who refused participation rather than being considered ineligible, after completing the first page of the survey but going no further. The assumption is being made that since we do not have definitive evidence as to the motive behind the survey break-off, we should estimate these unknowns to have the same refusal-to-ineligible proportions as those we heard from directly.

*2.20 Eligible, non-contact*

We assumed everyone in our email distribution list was eligible for the survey, given that they met our two eligibility criteria: 2) reported equal to or more than 200 potable service coonnections (SC) and 2) participated in the 2014 SWRCB Division of Drinking Water (DDW) annual reporting process;. Because we considered the SWRCB DDW’s email list to be “accurate and current, it can be assumed that all those from whom one receives no response are eligible sample persons who therefore must be treated as non-respondents” (AAPOR, 2015). Therefore we consider these selected respondents as eligible but unable to complete the questionnaire.

3.0 Unknown Eligibility, No Questionnaire Returned

*3.3 Email returned/Undeliverable*

We have presumed that contact list from the SWRCB includes eligible respondents based on an accurate and current data source. The instances in which the survey e-mail invitation generated a response that indicates the invitation was undelivered, is classified under an “unknown eligibility” category-- emails that bounced back (3.3).

# 2. Additional Explanation of Methods and Examination of Results

## 2.1 Preparedness Index Indicators

To build the climate preparedness index, we used eleven dummy variables to represent the three dimensions of preparedness evaluated (awareness, analytical capacity, and adaptation action). Variables were derived from survey responses, unless otherwise noted. To compute the score for *Awareness*, we combined four variables collected from the survey. These included agreement (1) or lack of agreement (neutral or disagreement, 0) that climate change is happening globally and locally, and agreement or lack of agreement that climate change is or will affect water quality globally and locally (Table S11). We calculated the standardized mean of the four variables to compare across each respondent.

We similarly computed *Analytic Capacity* and *Adaptation Action* across four and three variables, respectively. The indicators used to represent analytic capacity sought to represent the technical experience of the utility with modeling its own water system and the presence of a relationship with climate scientists. This sub-index is calculated with the underlying assumption that having such an operations model and communicating with climate scientists would likely increase a utilities’ capacity to technically examine the implications of and/or plan for climate change impacts. We also used the utility’s participation in two major water planning processes (Urban Water Management Plan and Integrated Regional Water Management Plan), both of which have guidance that strongly recommend the consideration of climate change impacts on water supplies.

For *Adaptation Action* we combined the self-reported action of respondent’s adaptation activities with their participation in locally-formed, voluntary regional climate collaboration organizations. To gauge the climate adaptation action, the survey asked participants specifically if they were engaged in any adaptation activities so far, including the early stages from discussing it or conducting a vulnerability assessment to implementing a strategy or other plan (based on heuristic of an adaptation process in Moser & Ekstrom 2009).

Table S11. Indicators used to construct for scores of each climate preparedness dimension

|  | **Indicator** | **Scale** | **Scale definitions** | **Normalization** |
| --- | --- | --- | --- | --- |
| **Awareness** | Agreement/disagreement that climate change is happening | 0,1 | 0=no (neutral or disagreement); 1=yes (any type of agreement) | No rescaling, used binary |
|  | Agreement/disagreement that climate change is/will affect global water quality | 0,1 | Same as above | No rescaling, used binary |
|  | Agreement/disagreement that climate change is happening in California | 0,1 | Same as above | No rescaling, used binary |
|  | Agreement/disagreement that climate change is/will affect local water quality | 0,1 | Same as above | No rescaling, used binary |
| **Analytical Capacity** | Have an operations model that can use climate input | 0,1,2 | 0=no; 1=operations model, no climate variables; 2= operations model that can take climate data | Transformed to 0, 0.5, 1 |
|  | Communicate with any climate change experts | 0,1 | 0=no; 1=yes | No rescaling, used binary |
|  | Have Urban Water Management Plan (required by size or volume of supply) | 0,1 | 0=no; 1=yes | No rescaling, used binary |
|  | Participant in Integrated Regional Water Management Plan | 0,1 | 0=no; 1=yes | No rescaling, used binary |
| **Climate Adaptation Action** | Presence/absence of respondent-reported climate adaptation activity | 0,1 | 0=none; 1=some action | No rescaling, used binary |
|  | Most advanced stage of reported adaptation activity | 0,1,2,3 | 0= None; 1=Understanding; 2=Planning; 3=Managing | Transformed to 0-1 |
|  | Participation in any regional collaborative process | 0,1 | 0=no; 1=yes | No rescaling, used binary |

## 2.2 Additional Summarized Survey Results


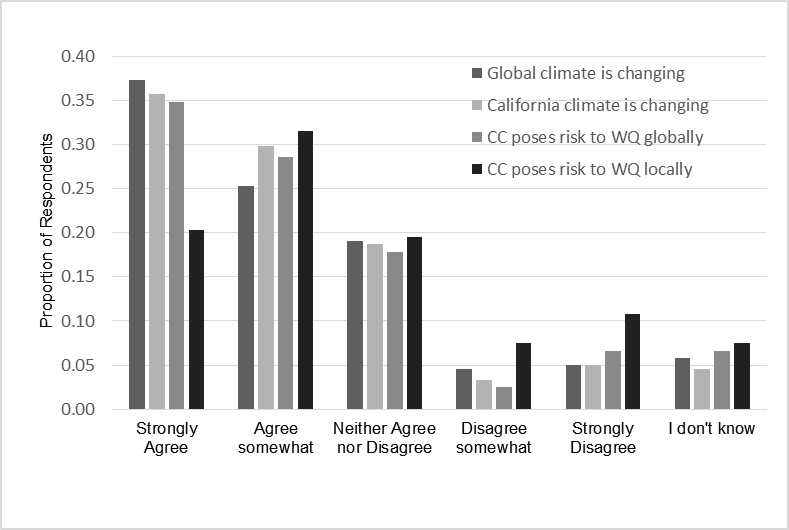


Figure S2. Bar charts show results of question “Please indicate your level of agreement with the following statements” (A) The global climate is changing; (B) California climate is changing; (C) Climate change poses risk to water quality globally; and (D) Climate change poses risk to water quality locally.”

Figure S3. Percent of utilities that have an operations model showed based on utility size (number of employees)

Figure S4. Climate preparedness dimensions summarized by water supply portfolio type. Some utilities are counted in more than one grouping because categories are not exclusive (e.g., a utility that has “only surface water” also fits in the category of “any surface water”

**Variables tested for association to climate adaptation activity**

We ran a series of bivariate Spearman’s Rho correlation tests between a utility’s most advanced self-reported phase of climate adaptation action (1-10) and the following variables, most of which were compiled from survey results. The purpose of the preliminary correlation tests was to identify significant variables to guide a more focused future investigation, which can be more tailored to investigating drivers of activities, traits, and other system attributes that may contribute to increasing adaptation advancement. Source of data and scoring applied noted in parentheses.

Awareness, Belief, and Perceived Risk

1. *Awareness Global*: Climate change belief and awareness: globally climate change is happening (scored 1-5, where 1= Strong disagreement, 2= Disagree somewhat, 3= Neither agree or disagree, 4= Somewhat agree, and 5= Strong agreement)
2. *Awareness Local:* Climate change belief and awareness: in California climate change is happening (scoring 1-5, same as above)
3. *Impact Global*: Climate change belief and awareness: climate change threatens water quality globally (scoring 1-5, same as above)
4. *Impact Local*: Climate change belief and awareness: climate change threatens local water quality (scoring 1-5, same as above)

Organizational Capacity

1. *Size*: Utility size (based on reported number of employees, 1= smallest with 1-10 employees to 6= largest with more than 500 employees)
2. *Pop Served*: Permanent population served (reported in SWRCB annual survey 2014, scalar)
3. *Regional Planning:* Regional planning process involvement (count of total processes reported by respondent, 0-7)
4. *Other Provisions*: Provision of other services beyond drinking water (count of total other services provided, 0-7)

Climate Information Analytical Capacity

1. *Climate Experts:* Variety of communication with climate experts (count of number of types organizations selected, 0-6)
2. *Operations Model:* Utility has an operations model (0 – no model; 0.5= has model but cannot take climate variables; 1.0 = has model that can use climate variables

Present Threats

1. *WQ Threat*: Count of present threats to water quality (score based on degree of severity reported – 1 to 5 for a series of 8 issues)
2. *Drought Impact:* - Reported degree to which the drought has impacted utility’s water quality (1= Not impacted water quality, 2= Slightly impacted water quality, 3= Somewhat seriously impacted water quality, 4= Seriously impacted water quality, 5= Very seriously impacted water quality)

Water Supply Source

1. *Surface water volume* (reported in SWRCB annual survey 2014, scalar)
2. *Groundwater volume* (reported in SWRCB annual survey 2014, scalar)
3. *Purchased water volume* (reported in SWRCB annual survey 2014, scalar)

Table S12. Correlation of ordinal variables that tested statistically significant at the p=0.05 level (two-tailed Spearman Rank); * are variables that are significant at the p=0.01 level. MAKE THIS TABLE SMALLER

| **Variable** | **Correlation Coefficient** | **Significance (2-tailed)** | **N** |
| --- | --- | --- | --- |
| Awareness Global | 0.15 | .035 | 196 |
| Awareness Local | 0.20 | .006 | 195 |
| Global Impact | 0.20 | .004 | 199 |
| Local Impact | 0.34 | <.000 | 194 |
| Climate Expert | 0.18 | .008 | 209 |
| Other Provisions | 0.18 | .010 | 209 |
| Drought Impact | 0.19 | .007 | 207 |
| Surface Water Volume | 0.21 | .002 | 209 |
| Regional Planning | 0.20 | .004 | 199 |

References

American Association for Public Opinion Research (AAPOR) (2015) Standard Definitions Final Dispositions of Case Codes and Outcome Rates for Surveys Available from: <http://www.aapor.org/AAPORKentico/AAPOR_Main/media/publications/Standard-Definitions2015_8theditionwithchanges_April2015_logo.pdf>

CEMA and CNRA (2012). California Adaptation Planning Guide: Understanding Regional

Characteristics http://www.ca-ilg.org/sites/main/files/fileattachments/

apg_understanding_regional_characteristics.pdf

Drinking Water Program (DWP) 2016. Drinking Water Information Clearinghouse: DRINC PORTAL, downloaded dataset from State Drinking Water Information System Public Water Systems (modified 4/8/16) at <http://drinc.ca.gov/dnn/Applications/DWPRepository.aspx>, Accessed 4/16/16.

Finzi Hart, J. A., P. M. Grifman, S. C. Moser, A. Abeles, M. R. Myers, S. C. Schlosser, J. A. Ekstrom (2012) Rising to the Challenge: Results of the 2011 Coastal California Adaptation Needs Assessment. USCSG-TR-01-2012. Available from: <https://woods.stanford.edu/sites/default/files/files/CACoastalAssessmentResults.pdf>

Moser, S.C, and J.A. Ekstrom (2010) A Framework to Diagnose Barriers to Climate Change Adaptation. PNAS 107(51):22026-22031.

State Water Resources Control Board (SWRCB) (2014). 2014 Annual Compliance Report. <http://www.waterboards.ca.gov/drinking_water/certlic/drinkingwater/documents/dwdocuments/2014/2014_acr_final.pdf>

Tribbia J, Moser SC (2008) More than information: what coastal managers need to plan for climate change. Environ Sci Policy 11:315–328. doi: 10.1016/j.envsci.2008.01.003

1. The summary by Climate Impact Region in this table is based on a more recent database of California PWS. The Drinking Water Division posted a list of active PWS in California dated April 8, 2016, available for download from <https://drinc.ca.gov/dnn/Applications/DWPRepository.aspx>. At the time of the survey, the state reported a total of 7789 PWS in their 2014 Annual Compliance Report (Table 1, <http://www.waterboards.ca.gov/drinking_water/certlic/drinkingwater/documents/dwdocuments/2014/2014_acr_final.pdf>) [↑](#footnote-ref-1)
2. AAPOR (2010) Response Rate Calculator. Available from: <https://www.aapor.org/AAPOR_Main/media/MainSiteFiles/ResponseRateCalculatorVer3-1_11_22_10.xls> [↑](#footnote-ref-2)
